# Connection between trajectory of primary cancer monitoring indicators and mortality after cancer in South Korea

**DOI:** 10.1186/s12916-025-04121-y

**Published:** 2025-05-30

**Authors:** Jung Hyun Kim, Haedong Kim, Man S. Kim, Mison Chun, Jaeyong Shin

**Affiliations:** 1 Division of Tourism and Wellness, Hankuk University of Foreign Studies (HUFS), Yongin, Republic of Korea; 2 School of Economics, Yonsei University, Seoul, Republic of Korea; 3 School of Medicine, Translational-Transdisciplinary Research Center, Clinical Research Institute, Kyung Hee University Hospital at Gangdong, Kyung Hee University, Seoul, Republic of Korea; 4 Department of Radiation Oncology, School of Medicine, Ajou University, Suwon, South Korea; 5 Department of Preventive Medicine and Public Health, College of Medicine, Yonsei University, 50-1 Yeonsei Ro, Seoul, Seodaemun-Gu 03722 Republic of Korea; 6 Institute for Innovation in Digital Healthcare, Yonsei University, Seoul, Republic of Korea

**Keywords:** Cancer prevention, Cancer case fatality, Prevention indicators, SDG3, Good health and well-being

## Abstract

**Background:**

Cancer remains a leading global cause of mortality, responsible for nearly 10 million deaths in 2020. Given the country’s low birth rate and aging population, the escalating cancer burden poses significant challenges to its healthcare systems. This study aimed to investigate the relationship between lifestyle risk factors and cancer case fatality, emphasizing the collective impact of these factors through a prevention index at the regional level.

**Methods:**

The study focused on ten cancer types, categorizing counties into three levels of cancer incidence rates using group-based trajectory modeling to identify disparities in patterns and levels among groups. Additionally, we segmented the proportions of obesity prevalence, average daily per capita smoking amount, prevalence of smoking, prevalence of high-risk alcohol consumption, prevalence of hypertension diagnosis, prevalence of diabetes diagnosis, and cancer case fatality into three groups through group-based trajectory modeling. Cox proportional hazard models were employed to evaluate the hazard ratios (HR) for cancer case fatality, adjusting for age, sex, income level, and cancer stage.

**Results:**

The study population comprised 294,070 cancer patients, with thyroid, stomach, colorectal, breast, and lung cancers being the most common. The prevention index (PI) levels, calculated from six primary prevention indicators, were categorized into High, Medium, and Low grades. Counties with higher PI levels (H) exhibited significantly lower cancer case fatality among cancer patients compared to those with lower PI levels (L). Across all cancer types, females had lower cancer case fatality compared to males, higher age was linked to higher cancer case fatality, advanced stage cases had the highest cancer case fatality, and the highest income quintile consistently showed the lowest cancer case fatality.

**Conclusions:**

The study highlights the significant inverse relationship between primary prevention indicator levels and cancer case fatality. Higher scores on primary prevention indicators are associated with lower cancer mortality among cancer patients for various cancer types, underscoring the importance of comprehensive, community-based prevention strategies in mitigating cancer risk and improving public health outcomes in South Korea.

**Supplementary Information:**

The online version contains supplementary material available at 10.1186/s12916-025-04121-y.

## Background

Cancer remains a leading global cause of mortality, accounting for nearly 10 million deaths in 2022 [1]. Approximately 20.3 million new cancer cases were reported worldwide in 2023. The most commonly diagnosed cancers globally include lung cancer (12.4% of new cases), breast cancer (11.6%), and colorectal cancer (9.6%), which collectively account for a significant proportion of the cancer burden. Lung cancer also remains the leading cause of cancer-related deaths, responsible for 18.7% of all cancer deaths, followed by colorectal and liver cancers​ [1]. The incidence of cancer has witnessed a significant surge over the span of five years, soaring from 1,239,171 cases in 2016 to 1,535,047 cases by 2021 in South Korea. Simultaneously, the mortality rate due to cancer has followed a similar upward trajectory, reaching 82,688 deaths within the same period. This escalating trend in cancer diagnoses and fatalities is especially worrying given situations that persistently low birth rate and the steady aging of its population in Korea [2–4]. These demographic factors indicate a potential exacerbation of the cancer burden in the years to come, posing considerable challenges to healthcare systems and public health initiatives. Therefore, preventing cancer and averting premature deaths resulting from it are critical priorities.

Much of cancer is preventable [5]. Cancer risk factors include smoking, excessive alcohol consumption, obesity, hypertension, and diabetes. In contrast, preventive factors involve maintaining a healthy weight, engaging in regular physical activity, adhering to a nutritious diet, and avoiding tobacco use and excessive alcohol intake. Addressing these risk factors and promoting preventive behaviors represent highly cost-effective strategies for both preventing and managing cancer [6, 7]. Investigating risk factors individually fails to capture their distribution in the population adequately. Cancer is influenced by multiple lifestyle risk factors, which often co-occur simultaneously [8, 9]. Additionally, these risk factors have been demonstrated to act synergistically in the progression of cancer [10, 11]. Therefore, it is imperative to evaluate the collective impact of lifestyle factors on health outcomes to gain a deeper understanding of their association with health outcomes.

Prior research has indicated an association between primary cancer indicators and mortality from total cancer as well as specific types of cancer mortality [12–15]. Our objective was to explore this relationship within a general population sample from Korea and to reinforce the current evidence suggesting that overall lifestyle by regional level, as indicated by a prevention index level, influences the risk of cancer mortality. We evaluated the association between six primary prevention indicators and the ten specific cancer mortality: thyroid, breast, colorectal, cervical, stomach, lung, prostate, pancreatic cancers, non-Hodgkin lymphomas, and leukemia. These cancers were selected due to their prevalence in South Korea and their potential association with modifiable lifestyle risk factors. In our main analysis, we sought to determine whether adherence to the cancer prevention guidelines was linked to a decreased risk of mortality from specific types of cancer. Trajectory analysis is a method recently embraced in epidemiology for monitoring temporal behavior patterns [16, 17]. To our knowledge, no studies have examined combined regional level primary cancer indicator trajectories following a cancer diagnosis thus far. Consequently, this study aimed to assess the relationship between lifestyle risk factor distribution and mortality risk, utilizing trajectory analysis to comprehensively capture lifestyle risk patterns post-cancer diagnosis.

## Methods

### Study design and setting

This study aimed to evaluate the relationship between primary cancer prevention indicators, cancer incidence, and cancer case fatality, in 42 counties of Gyeonggi Province, South Korea. The analysis used data from 2010 to 2018 for prevention indicators, and up to 2020 for cancer incidence and mortality, integrating group-based trajectory modeling and Cox proportional hazard ratio analysis. The dependent variable in this study was cancer case fatality, derived from the National Cancer Center Korea and National Health Insurance Service (NHIS) databases. Cancer incidence was based on pathology-confirmed diagnoses and classified according to International Classification of Diseases (ICD) codes (Additional file 1: Table S1). Independent variables were composite scores (three prevention index levels) of six primary prevention indicators (obesity, high-risk alcohol consumption, prevalence of smoking, daily smoking amount, hypertension, and diabetes prevalence), and age, sex, cancer stage, and income quintiles were adjusted as covariates. This study combines ecological and cohort methodologies to examine the association between county-level prevention efforts and individual-level cancer outcomes. The ecological component evaluates patterns in prevention indicators and cancer incidence rates across counties, while the cohort component assesses cancer case fatality, using individual-level data. The integration of these approaches enables the study to explore how community-level prevention efforts influence individual outcomes.

**Fig. 1:**
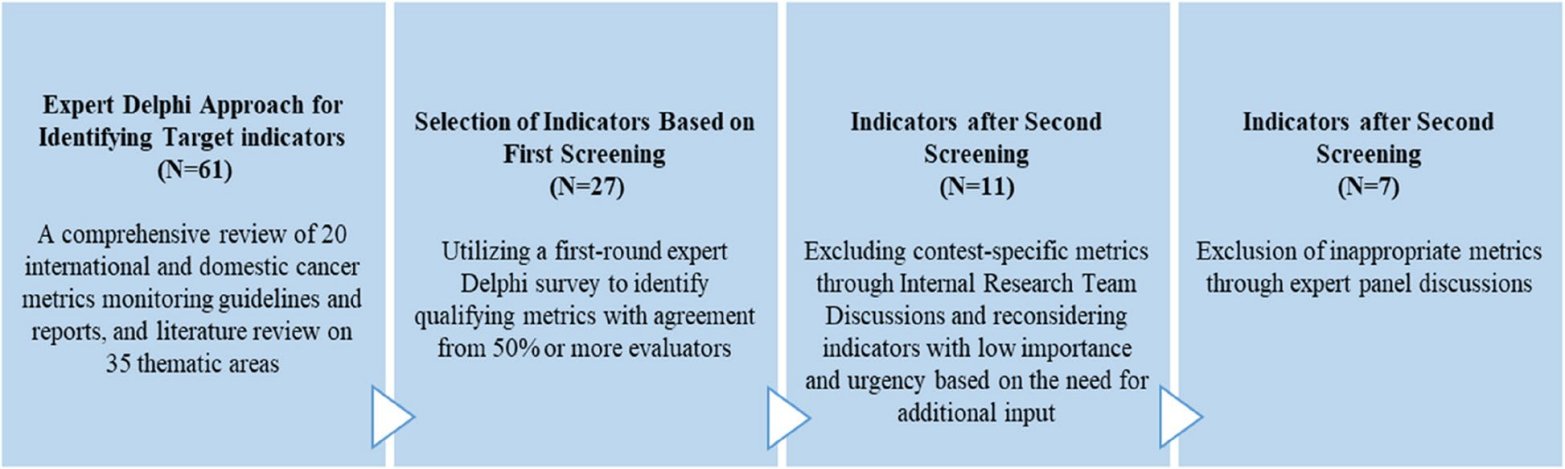
Selection process of primary preventive indicators for cancer monitoring

**Fig. 2:**
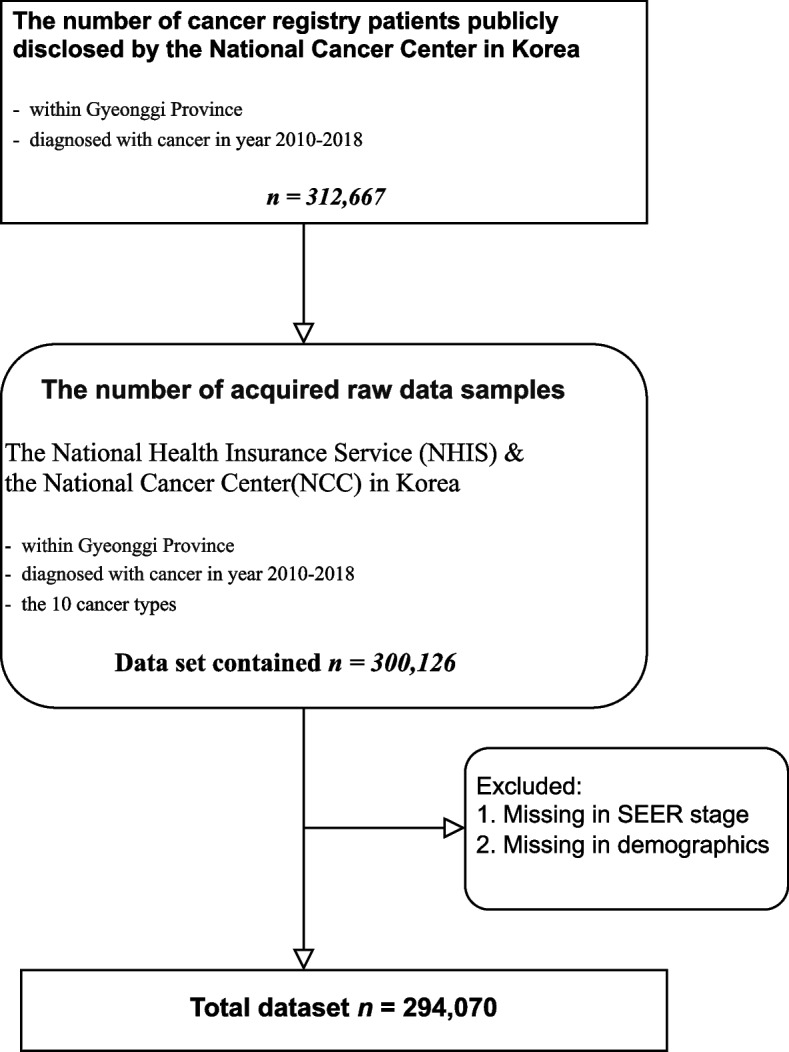
Flow chart of study population

In this study, we focused on evaluating primary prevention indicators aimed at monitoring and reducing cancer risk before its onset. To achieve this, we built upon the findings of our previous research, which provided valuable insights into the selection and relevance of these indicators [18]. In summary, we identified 61 indicators pertaining to primary prevention, covering various subdomains including obesity, physical activity, research and investment, alcohol consumption, smoking, nutrition, high risk of infection, high risk of chronic disease, high risk of occupational and environmental factors, medical care systems, health professionals, and vaccination and immunizations. These indicators were identified through a combination of methods including literature review, expert Delphi survey, and panel discussions (Fig. 1). After the three-screening process, a total of seven preventive indicators were selected for further analysis. Figure 2 presents a flowchart describing the population included in this study. (Fig. 2).

Table 1 presents a list of 7 region-specific cancer primary monitoring indicators. The detailed definitions of the indicators are provided in Additional file 1: Table S2. The indicators were computed at the local community level including counties.

**Table 1:** List of primary prevention indicators and its basic statistics from 2010–2018 in Gyeonggi Province

Primary prevention	Sub-domains	Indicators	Unit	Year	Mean (Std)
1	Obesity	Obesity Prevalence	Counties	2010–2018	27.6 (6.0)
2	Physical activity	Prevalence of Physical Activity Participation		2010–2017	20.4 (4.1)
3	Alcohol	Prevalence of High-Risk Alcohol Consumption		2010–2018	18.0 (3.8)
4	Risk for chronic disease and occupational environment	Prevalence of Hypertension Diagnosis		2010–2018	16.0 (1.5)
5	Risk for chronic disease and occupational environment	Prevalence of Diabetes Diagnosis		2010–2018	6.2 (1.1)
6	Smoking	Average Daily Smoking Consumption Per Capita		2010–2018	15.1 (1.1)
7	Smoking	Prevalence of Smoking		2010–2018	23.3 (3.5)

### Data sources and study population

The Korea Community Health Survey (KCHS) is a collaborative effort between the Korea Disease Control and Prevention Agency (KDCA) and 255 public health centers. Its objective is to assess the health status of residents in accordance with the law. Since its inception in 2008, this survey has been targeting approximately 900 individuals aged 19 and above per public health center [19]. The survey encompasses a variety of topics related to health behaviors and prevention, which is used to assess the prevalence of personal health practices and behaviors related to the leading causes of disease, including smoking, alcohol use, drinking and driving, high blood pressure control, physical activity, weight control, quality of life, medical service, accident, injury, etc. The KCHS was utilized to construct preventive indicator values for the 42 counties of Gyeonggi province, which is the largest population in South Korea, from 2010 to 2018. However, among the selected 7 preventive indicators, as shown in Table 1, data for physical activity prevalence in recent years could not be obtained. Consequently, this indicator was excluded, resulting in the final derivation of 6 primary preventive indicators.

The National Health Information Database (NHID), operated by the NHIS, is a publicly accessible database covering the entire South Korean population, which exceeds 50 million people. This database contains regularly updated information on sociodemographic factors, healthcare usage, mortality rates, and health screening data collected biennially for both insured individuals and their dependents [20]. In addition, information regarding the stage of cancer was obtained from the National Cancer Registration of the National Cancer Center Korea and combined with all datasets for the study.

### Cancer types, covariates, and county grouping

The incidence of 10 cancer types including thyroid cancer, breast cancer, colorectal cancer, cervical cancer, stomach cancer, lung cancer, prostate cancer, pancreatic cancer, non-hodgkin lymphomas, and leukemia were tracked from 2008 to 2020. These cancer incidences were then classified into 3 levels of cancer incidence rate per 100,000 persons using group-based trajectory modeling. To support the development of tailored community-based cancer management policies, this study conducted an analysis by assigning scores to groups based on prevention indicators. Consequently, the six primary prevention indicator values were utilized to determine cancer prevention excellence grades for each county. Age, sex, cancer stage, and income quintiles were adjusted as covariates.

### Statistical analysis

#### Step 1: Assessment of primary cancer prevention indicators

The first step focused on evaluating six primary cancer prevention indicators, including obesity, high-risk alcohol consumption, prevalence of smoking, smoking amount, hypertension, and diabetes. These indicators were analyzed longitudinally from 2010 to 2018 using group-based trajectory modeling, which identifies distinct patterns over time. Each indicator was classified into two or three trajectory groups based on observed patterns: indicators with limited variability (e.g., prevalence of smoking) were divided into two groups, with scores ranging from 1 (low performance) to 2 (high performance). Indicators with greater variability (e.g., hypertension) were divided into three groups, with scores ranging from 1 to 3. These scores were equally weighted and summed to calculate a composite score for each county, ranging from 6 to 15. The composite scores were then categorized into three prevention index levels: Low (6–8), Medium (9–11), and High (12–15). These thresholds were informed by the score distribution across districts, ensuring meaningful distinctions between performance levels. The grading system facilitated comparisons and highlighted regions requiring targeted interventions. These levels reflect the overall effectiveness of primary cancer prevention efforts in each district, with"High"indicating the strongest performance and"Low"highlighting regions needing improvement. Trajectory analysis employs semi-parametric group-based modeling techniques to recognize possible trends within complete sets of data. Each model symbolizes an individual exhibiting a comparable trajectory [21]. The best model fit was evaluated using the Bayesian Information Criterion (BIC), with the constraint that the number of trajectory groups be limited to fewer than six [22, 23]. This categorization enabled a comprehensive assessment of primary prevention performance across the districts.

#### Step 2: Analysis of cancer incidence patterns

The second step involved calculating regional cancer incidence rates for 10 cancer types: thyroid, breast, colorectal, cervical, stomach, lung, prostate, pancreatic cancers, non-Hodgkin lymphomas, and leukemia. Using the NHIS claims data, incidence rates were measured at the county level per 100,000 persons. Group-based trajectory modeling was again applied to categorize 42 counties into three incidence levels: High, Medium, and Low. This classification facilitated a comparison between cancer incidence levels and prevention index levels, enabling an evaluation of the relationship between prevention efforts and cancer incidence trends. Variations in regional cancer registration practices, screening activities, and healthcare access were acknowledged as potential confounders affecting cancer incidence rates.

#### Step 3: Statistical analysis of cancer case fatality

The third step utilized Cox proportional hazard modeling to analyze the impact of primary prevention indicators on cancer case fatality. The death of the cancer patients was followed from the first date of their first cancer diagnosis to the earliest event of death, emigration or end of the study follow-up of in 2020. Individual-level longitudinal data set was used, which included variables such as age, sex, SEER stage at diagnosis, and income quintiles. Intercorrelations between these variables, such as the association between obesity and hypertension, were assessed using variance inflation factors to address multicollinearity in the statistical models. Additionally, the latency periods between independent and dependent variables were considered by aligning longitudinal data to ensure sufficient temporal gaps. The prevention index levels (from Step 1) was incorporated into the Cox proportional hazards models as independent variables. This statistical approach allowed the study to assess the significance of primary prevention indicators while accounting for individual demographic and clinical factors, providing insights into how prevention efforts at the county level influence patient outcomes. Hazard ratios were considered statistically significant when the associated 95% confidence intervals (CIs) did not include unity, corresponding to a statistical test on the two-sided 5% significance level.

## Results

### Prevention index based on grouped trajectory modeling

Group-based trajectory modeling was employed investigating disparities in patterns and levels among various groups and drawing out their implications. The analysis unveiled that obesity prevalence, smoking amount, and prevalence of smoking were segmented into two groups, while prevalence of alcohol consumption, hypertension, diabetes, and cancer incidence rate were classified into three groups through statistical analysis.

**Fig. 3:**
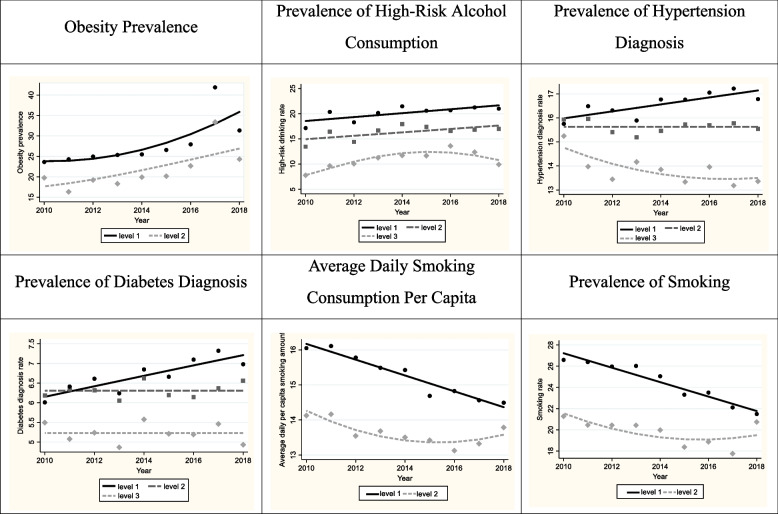
Cancer incidence trajectories by each preventive indicators: obesity prevalence, prevalence of high-risk alcohol consumption, prevalence of hypertension diagnoses, prevalence of diabetes diagnoses, average daily smoking consumption per capita, and prevalence of smoking from 2010–2018 in Gyeonggi Province

These proportions were classified into three levels using group-based trajectory modeling, as illustrated in Fig. 3, which depicts the trajectory of different patterns of risk factors per 100,000 persons. The second panel of the figure shows that level 1 exhibits the highest high-risk alcohol consumption rate, while level 3 represents the lowest rate among the groups. Similarly, three trajectories were distinguished in terms of cancer incidence rates among 42 counties during the period 2010–2018. The results showed that although average cancer incidence rates increased across all three groups, the levels varied significantly. In this study, the group with the lowest average cancer incidence rate was assigned a score of 3, while the group with the highest rate was assigned a score of 1. As observed in Fig. 3, level 1 exhibits the highest cancer incidence rate, while level 3 represents the lowest cancer incidence rate among the groups.

The 6 primary prevention indicator values were utilized to categorize the cancer prevention excellence grades for each county (Table 2, Additional file 1: Fig. S1) The composite score was calculated as the sum of the scores for the 6 primary prevention indicators, ranging from 6 to 15. Among the 42 counties, 4 were classified as H (High), 10 as M (Medium), and 28 as L (Low) grades of PI levels. For counties with the highest cancer incidence rate (CI level), it is notable that the PI level consistently remained the lowest, denoted as"L".

**Table 2:** Composite scores of primary prevention indicator values, prevention index level, and cancer incidence levels by each county during 2010–2020 in Gyeonggi Province

Counties	Obesity (2)	Alcohol (3)	Hypertension (3)	Diabetes (3)	Average daily smoking consumption (2)	Smoking amount (2)	Composite score (15)	PI level (3)	CI level (3)
GA	2	3	3	3	2	2	15	H	H
KA	2	3	3	3	2	2	15	H	M
GB	1	2	3	3	2	2	13	H	H
IA	1	2	2	3	2	2	12	H	H
AA	1	2	2	3	1	2	11	M	H
AB	1	2	2	3	1	2	11	M	H
KB	1	2	2	3	1	2	11	M	H
KC	1	2	2	3	1	2	11	M	H
GC	1	2	2	3	1	2	11	M	H
KD	1	2	2	3	1	2	11	M	M
EA	1	2	2	2	1	1	9	M	M
EB	1	2	2	2	1	1	9	M	M
AC	1	2	1	2	2	1	9	M	M
JA	1	2	2	2	1	1	9	M	M
JB	1	2	2	1	1	1	8	L	M
GD	1	1	2	2	1	1	8	L	M
GE	1	1	2	2	1	1	8	L	M
AD	1	2	1	2	1	1	8	L	M
IB	1	2	1	2	1	1	8	L	M
CA	1	1	2	2	1	1	8	L	M
FA	1	2	1	2	1	1	8	L	L
EC	1	1	1	2	1	1	7	L	L
IC	1	1	2	1	1	1	7	L	H
ED	1	1	2	1	1	1	7	L	L
GF	1	1	2	1	1	1	7	L	M
ID	1	2	1	1	1	1	7	L	M
KE	1	1	1	2	1	1	7	L	M
CB	1	1	1	2	1	1	7	L	M
B	1	2	1	1	1	1	7	L	M
IE	1	2	1	1	1	1	7	L	H
CC	1	1	1	1	1	1	6	L	L
D	1	1	1	1	1	1	6	L	M
LA	1	1	1	1	1	1	6	L	H
FB	1	1	1	1	1	1	6	L	M
HA	1	1	1	1	1	1	6	L	M
CD	1	1	1	1	1	1	6	L	M
GG	1	1	1	1	1	1	6	L	H
IF	1	1	1	1	1	1	6	L	H
LB	1	1	1	1	1	1	6	L	H
LC	1	1	1	1	1	1	6	L	H
HB	1	1	1	1	1	1	6	L	M
GH	1	1	1	1	1	1	6	L	M

County names are abbreviated to protect the privacy of the regions included in this analysis

#### General characteristics of the study population and cancer

Study participants were on average 58.7 (15.3) years. Among 294,070 cancer patients, the five leading cancer types were thyroid (22.1%), stomach (17.7%), colorectal (16.5%), breast (13.8%), and lung (13.1%) cancers. Lung, pancreatic, and leukemia were diagnosed at a later stage. The mean age of cancer diagnosis was youngest for thyroid cancer (46.5 years old) and oldest for prostate cancer (70.2 years old) (Table 3, Additional file 1: Table S3). In this study, the SEER classification was divided into four categories: Localized, Regional, Distant and Unknown, while income levels were divided into five quintiles, each representing 20% of the population.

**Table 3:** General characteristics of the study population at the time of cancer diagnosis by cancer types and prevention index level during 2010–2020 in Gyeonggi Province

	Thyroid cancer (*N* = 65,070)				Breast cancer (*N* = 40,596)					
	High (*N* = 7665)	Medium (*N* = 20,936)	Low (*N* = 36,469)		High (*N* = 4387)	Medium (*N* = 13,223)			Low (*N* = 22,986)	
Age, mean (SD)	45.1 (11.5)	47.1 (11.9)	47.4 (12.2)		51.6 (11.7)	51.8 (11.2)			52.2 (11.4)	
SEER General Summary Staging system, N(%)										
Localized	2733 (35.7)	8304 (39.7)		14,893 (40.8)	2716 (61.9)	7767 (58.7)			13,305 (57.9)	
Regional	4238 (55.3)	11,069 (52.9)		19,211 (52.7)	1341 (30.6)	4500 (34.0)			7868 (34.2)	
Distant	35 (0.46)	117 (0.56)		246 (0.67)	199(4.5)	585 (4.4)			1126 (4.9)	
Unknown	659 (8.6)	1446 (6.9)		2119 (5.8)	131 (3.0)	371 (2.8)			687 (3.0)	
Income, mean (SD)	4 (1.3)	3.5 (1.4)		3.4 (1.4)	3.8 (1.4)	3.3 (1.5)			3.1 (1.5)	
	Colorectal cancer (*N* = 48,500)				Cervical cancer (*N* = 6,823)					
	High (*N* = 3810)	Medium (*N* = 14,690)	Low (*N* = 30,000)		High (*N* = 490)		Medium (*N* = 1994)			Low (*N* = 4339)
Age, mean (SD)	63.7 (14.6)	63.7 (13.2)	64.4 (15.5)		51.0 (15.5)		51.8 (14.6)	52.9 (15.0)		
SEER General Summary Staging system, N(%)										
Localized	1492 (39.2)	5935 (40.4)	11,226 (37.4)		275 (56.1)		1150 (57.7)	2488 (57.3)		
Regional	1553 (40.8)	5683 (38.7)	12,411 (41.4)		115 (23.5)		528 (26.5)	1159 (26.7)		
Distant	553 (14.5)	2262 (15.4)	4668 (15.6)		46 (9.4)		166 (8.3)	379 (8.7)		
Unknown	212 (5.6)	810 (5.5)	1695 (5.7)		54 (11.0)		150 (7.5)	313 (7.2)		
Income, mean (SD)	3.7 (1.5)	3.3 (1.5)	3.2 (1.5)		3.4 (1.5)		3.0 1.5)	2.9 (1.4)		
	Stomach cancer (*N* = 51,941)				Lung cancer (*N* = 38,383)					
	High (*N* = 4137)	Medium (*N* = 15,067)	Low (*N* = 32,737)		High (*N* = 3002)		Medium (*N* = 10,804)			Low (*N* = 24,577)
Age, mean (SD)	62.5 (13.6)	62.5 (12.7)	63.2 (12.8)		67.3 (12.6)	68.2 (11.7)			68.5 (11.4)	
SEER General Summary Staging system, N(%)										
Localized	2625 (63.5)	9732 (64.6)	20,299 (62.0)		900 (30.0)	2459 (22.8)			5408 (22.0)	
Regional	902 (21.8)	3004 (19.9)	7257 (22.2)		786(26.2)	2782 (25.7)			6774 (27.6)	
Distant	415 (10.0)	1575 (10.5)	3590 (11.0)		1035 (34.5)	4620 (42.8)			10,301 (41.9)	
Unknown	195 (4.7)	756 (5.0)	1591 (4.9)		281 (9.4)	943 (8.7)			2094 (8.5)	
Income, mean (SD)	3.8 (1.5)	3.3 (1.5)	3.2 (1.5)		3.8 (1.5)	3.3 (1.5)			3.2 (1.5)	
	Prostate cancer (*N* = 18,774)				Pancreatic cancer (*N* = 10,136)					
	High (*N* = 2315)	Medium (*N* = 5636)	Low (*N* = 10,823)		High (*N* = 838)		Medium (*N* = 3066)			Low (*N* = 6232)
Age, mean (SD)	70.2 (9.0)	70.1 (8.8)	70.4 (8.5)		67.5 (14.1)		67.3 (13.1)			67.5 (12.5)
SEER General Summary Staging system, N(%)										
Localized	1306 (56.4)	3334 (59.2)	6257 (57.8)		102 (12.2)		327 (10.7)			811 (13.0)
Regional	440 (19.0)	1169 (20.7)	2274 (21.0)		252 (30.1)		979 (31.9)			1915 (30.7)
Distant	133 (5.7)	534 (9.5)	989 (9.1)		376 (44.9)		1406 (45.9)			2751 (44.1)
Unknown	436 (18.8)	599 (10.6)	1303 (12.0)		108 (12.9)		354 (11.5)			755 (12.1)
Income, mean (SD)	4 (1.4)	3.6 (1.5)	3.5 (1.5)		3.9 (1.4)		3.4 (1.5)			3.2 (1.5)
	Non-Hodgkin lymphomas (*N* = 7,929)				Leukemia (*N* = 5,918)					
	High (*N* = 816)	Medium (*N* = 2424)	Low (*N* = 4689)		High (*N* = 533)		Medium (*N* = 1790)			Low (*N* = 3595)
Age, mean (SD)	58.3 (18.9)	56.2 (19.6)	57.0 (19.5)		46.4 (23.4)		48.1 (23.3)			49.1 (23.1)
SEER General Summary Staging system, N(%)										
Localized	218 (26.7)	744 (30.7)	1433 (30.6)		-		-			-
Regional	101 (12.4)	389 (16.0)	608 (13.0)		-		-			-
Distant	308 (37.7)	937 (38.7)	1976 (42.1)		505 (94.7)		1686 (94.2)			3428 (95.4)
Unknown	189 (23.2)	354 (14.6)	672 (14.3)		28 (5.3)		104 (5.8)			167 (4.6)
Income, mean (SD)	3.9 (1.4)	3.5 (1.5)	3.3 (1.5)		3.9 (1.4)		3.4 (1.4)			3.3 (1.5)

**Fig. 4:**
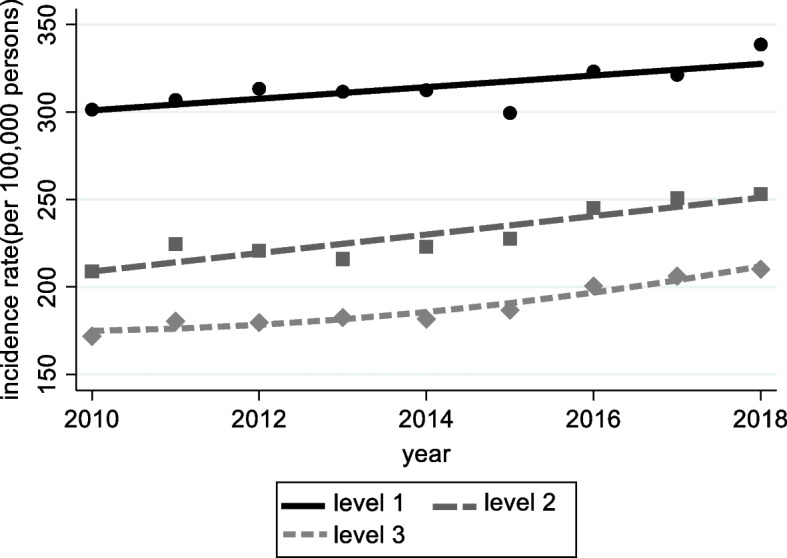
Trajectories in terms of cancer incidence rates among 42 counties, during the period 2010–2018 in Gyeonggi Province

In Fig. 4, it represents the trajectory model analysis, showing the different levels of cancer incidence rate per 100,000 persons. As observed in the figure, Level 1 exhibits the highest cancer incidence rate, while Level 3 represents the lowest cancer incidence rate among the groups.

To examine the relationship between PI level and the total mortality among the cancer patients, Hazard Ratio analysis was conducted for the 10 cancer types. Gender, age, SEER stage, income quintiles, and PI level were utilized as explanatory variables. In terms of gender, males served as the reference category, SEER stage utilized stage 1 as the reference category, income quintiles used the 1 st quintile as the reference category, and for PI level,'L'served as the reference category. The analysis results are presented in Table 4. The variable of interest, PI level, exhibited a significant inverse relationship with the case fatality among cancer patients. Specifically, when PI level was at the highest grade (H), the case fatality was significantly lower compared to the lowest grade (L) for all the cancer sites except for cervical cancer. The findings indicate varying degrees of disparity in the case fatality rates, with non-overlapping confidence intervals between high and medium PI levels for colorectal, stomach, lung, prostate, and non-Hodgkin lymphoma. Across all cancer types, females exhibited relatively lower case fatality compared to males, while higher age was consistently associated with higher mortality. Notably, cases with advanced stage displayed the highest case fatality. Regarding income, minimal variation was observed among the 2nd to 4 th quintiles; however, the highest income quintile (5 th) consistently demonstrated the lowest case fatality for all cancer types.

**Table 4:** Relative risks of cancer case fatality by cancer types and primary prevention indicator level during 2010–2020 in Gyeonggi Province

	Thyroid Cancer	Breast Cancer	Colorectal Cancer	Cervical cancer	Stomach cancer
Age	1.12*** (1.11 ~ 1.12)	1.04*** (1.04 ~ 1.04)	1.06*** (1.06 ~ 1.06)	1.05*** (1.04 ~ 1.05)	1.05*** (1.05 ~ 1.05)
Sex					
Female	0.53*** (0.47 ~ 0.59)	0.83 (0.59 ~ 1.17)	0.91*** (0.88 ~ 0.93)		0.92*** (0.89 ~ 0.95)
SEER stage					
Regional	1.19*** (1.05 ~ 1.34)	2.81*** (2.61 ~ 3.03)	1.77*** (1.70 ~ 1.85)	2.69*** (2.37 ~ 3.05)	3.97*** (3.82 ~ 4.13)
Advanced	10.96*** (9.13 ~ 13.17)	24.10*** (22.22 ~ 26.13)	10.49*** (10.04 ~ 10.95)	10.87*** (9.49 ~ 12.44)	22.10*** (21.21 ~ 23.03)
Unknown	2.61*** (2.22 ~ 3.07)	5.14*** (4.51 ~ 5.85)	3.36*** (3.15 ~ 3.57)	3.12*** (2.63 ~ 3.72)	5.72*** (5.41 ~ 6.05)
Income quintiles					
2	0.70*** (0.58 ~ 0.85)	0.89** (0.80 ~ 0.97)	0.89*** (0.84 ~ 0.93)	0.95 (0.82 ~ 1.11)	0.98 (0.93 ~ 1.03)
3	0.78*** (0.65 ~ 0.93)	0.85*** (0.77 ~ 0.93)	0.89*** (0.84 ~ 0.93)	0.89 (0.77 ~ 1.04)	0.94*** (0.89 ~ 0.98)
4	0.73*** (0.62 ~ 0.85)	0.81*** (0.74 ~ 0.89)	0.89*** (0.85 ~ 0.93)	0.86** (0.74 ~ 0.99)	0.91*** (0.87 ~ 0.95)
5	0.58*** (0.50 ~ 0.66)	0.72*** (0.66 ~ 0.78)	0.79*** (0.76 ~ 0.83)	0.74*** (0.65 ~ 0.86)	0.81*** (0.78 ~ 0.85)
PI level					
High	0.82** (0.68 ~ 0.99)	0.86*** (0.78 ~ 0.96)	0.81*** (0.76 ~ 0.86)	1.01 (0.83 ~ 1.23)	0.84*** (0.79 ~ 0.89)
Medium	0.91* (0.81 ~ 1.01)	0.88*** (0.82 ~ 0.94)	0.93*** (0.90 ~ 0.96)	0.96 (0.86 ~ 1.07)	0.93*** (0.90 ~ 0.96)
	Lung Cancer	Prostate Cancer	Pancreatic cancer	Non-Hodgkin lymphomas	Leukemia
Age	1.04*** (1.04 ~ 1.04)	1.09*** (1.09 ~ 1.10)	1.03*** (1.03 ~ 1.03)	1.05*** (1.04 ~ 1.05)	1.03*** (1.03 ~ 1.04)
Sex					
Female	0.65*** (0.63 ~ 0.67)		0.90*** (0.87 ~ 0.94)	0.83*** (0.77 ~ 0.89)	0.90*** (0.84 ~ 0.97)
SEER stage					
Regional	2.06*** (1.98 ~ 2.15)	1.20*** (1.10 ~ 1.30)	1.39*** (1.29 ~ 1.51)	1.025 (0.90 ~ 1.15)	-
Advanced	5.66*** (5.45 ~ 5.88)	6.07*** (5.65 ~ 6.53)	3.40*** (3.15 ~ 3.67)	1.70*** (1.56 ~ 1.85)	-
Unknown	3.12*** (2.96 ~ 3.28)	1.52*** (1.40 ~ 1.65)	1.70*** (1.56 ~ 1.87)	1.42*** (1.27 ~ 1.59)	-
Income quintiles					
2	0.98 (0.94 ~ 1.02)	0.97 (0.87 ~ 1.08)	0.90*** (0.83 ~ 0.97)	0.95 (0.84 ~ 1.07)	0.92 (0.81 ~ 1.05)
3	0.91*** (0.88 ~ 0.95)	1.02 (0.92 ~ 1.13)	0.93** (0.86 ~ 1.00)	0.91 (0.80 ~ 1.02)	0.93 (0.82 ~ 1.06)
4	0.88*** (0.84 ~ 0.91)	0.89** (0.81 ~ 0.98)	0.89*** (0.84 ~ 0.95)	0.94 (0.84 ~ 1.05)	0.90* (0.80 ~ 1.01)
5	0.78*** (0.76 ~ 0.81)	0.78*** (0.72 ~ 0.84)	0.86*** (0.81 ~ 0.91)	0.83*** (0.75 ~ 0.92)	0.83*** (0.74 ~ 0.92)
PI level					
High	0.75*** (0.72 ~ 0.79)	0.67*** (0.60 ~ 0.74)	0.86*** (0.79 ~ 0.93)	0.73*** (0.65 ~ 0.83)	0.86** (0.75 ~ 0.99)
Medium	0.92*** (0.89 ~ 0.94)	0.93** (0.87 ~ 0.99)	0.94*** (0.90 ~ 0.98)	0.94* (0.87 ~ 1.01)	0.92** (0.85 ~ 1.00)

Adjusted for age, sex, SEER stage, and income level

The symbols *(*p* <.1), **(*p* <.05) and ***(*p* <.01) indicate statistical significance, and the confidence intervals in parentheses represent 95%

## Discussion

In this study, our objective is to analyze the trajectories of six primary prevention indicators across 42 counties in Gyeonggi Province, South Korea, using group-based trajectory modeling and to explore the relationship between a comprehensive set of prevention indicator levels at the regional level and risk of site-specific cancer case fatality. Using trajectory analysis, this study revealed a more comprehensive profile of levels of primary prevention patterns among cancer patients when various prevention indicators were considered simultaneously. Incorporating a range of healthy lifestyle habits led to a notable decrease in the likelihood of cancer-related deaths. Adhering to healthy lifestyles was associated with lower risks of several cancer types, including thyroid, breast, colorectal, cervical, stomach, lung, prostate, pancreatic cancers, non-Hodgkin lymphomas, and leukemia. The adoption of the healthiest lifestyle practices was associated with a lower risk of cancer mortality among the cancer patients ranging from 14 to 33% reduction for the 9 sites, but none for cervical cancer compared to individuals with the lowest level of preventive indicators. For lung and prostate cancers and, non-Hodgkin lymphoma, the differences between these groups were relatively modest, suggesting similar outcomes for higher-performing regions. In contrast, for colorectal and thyroid cancers, the disparities were more substantial, underscoring the impact of achieving the highest level of preventive indicators. Interestingly, for breast cancer, no significant difference in case fatality was observed between H and M regions, highlighting potential biological or treatment-related factors that mitigate the influence of prevention levels. For stomach and pancreatic cancers, the disparities between H and M regions were moderate but still meaningful, emphasizing the continued benefit of enhanced preventive measures.

Cancer prevention encompasses primary, secondary, and tertiary approaches. Primary prevention entails adopting healthy behaviors to decrease the likelihood of developing cancer [24, 25]. This research identified 6 indicators associated with primary prevention, focusing on areas such as obesity, alcohol consumption, smoking amount and prevalence, chronic disease risk including diabetes and hypertension. The Center for Disease Control recommends a healthy diet and regular moderate or vigorous physical activity [26]. Some studies emphasize empowering individuals through education to make healthier lifestyle choices regarding tobacco use and nutrition [27, 28]. The International Agency for Research on Cancer (IARC) has reported a correlation between obesity and increased cancer risk, including postmenopausal breast cancer, colon cancer, endometrial cancer, esophageal cancer, and kidney cancer.

We examined the relationships between combined lifestyle factors and the risk of various cancer types, observing that some cancers appear more susceptible to lifestyle influences than others. Notably, stronger associations were found with more aggressive cancers, such as colorectal, stomach, and lung cancer, compared to less aggressive types, such as thyroid, lymphoma, and cervical cancer. While the precise mechanisms remain unclear, it is conceivable that lifestyle factors exert a greater impact on more aggressive cancers due to their distinct underlying causes [29, 30]. However, it's important to note that certain risk factors were not accounted for in the lifestyle score when examining specific types of cancer. For example, second-hand smoking and air pollution were not factored into the scores for lung cancer, and factors such as endogenous and exogenous estrogen exposure history were not considered in the scores for breast cancer.

The relationships between individual healthy lifestyle factors and cancer risk have been extensively established. For instance, meta-analyses have demonstrated a dose–response connection between alcohol consumption and cancer risk: consuming 50 g and 100 g of ethanol per day was associated with 22% and 91% higher risks of developing cancer compared to abstainers [31], and heavy drinkers faced a 31% higher risk of cancer mortality compared to non-drinkers [32]. Body weight was similarly linked to various site-specific cancers: each five-unit increase in body mass index correlated with 5–50% higher risks of postmenopausal breast, colon and rectal, endometrial, esophageal, gallbladder, kidney, liver, ovarian, pancreatic, stomach cardia, and thyroid cancer, as well as meningioma and multiple myeloma [33]. Moreover, individuals with obesity had 6% and 10% higher risks of cancer mortality compared to those with normal weight [34]. Regarding physical activity and diet, individuals in the highest category had 9%−42% and 10% lower risks of cancer [35, 36], and 20% and 22% lower risks of cancer mortality, respectively, compared to those in the lowest category [37, 38]. Lastly, tobacco smoking stands out as the most significant risk factor for cancer morbidity and mortality. Current smokers faced a substantial increase in the risk of incident cancer, particularly for cancers of the lung, larynx, pharynx, upper digestive tract, and oral regions [39]. Furthermore, smokers experienced significantly heightened risks for both smoking-related cancers and other types of cancers [40].

Our analyses revealed that the associations between combined lifestyle factors and cancer case fatality remained largely consistent across various socioeconomic backgrounds. These backgrounds include different age groups, genders, geographic regions, and economic levels. Therefore, it's essential for each country and region to develop policies that are customized to the preferences of the local population and the realities of local public health practices. This approach is crucial for advancing progress towards achieving Sustainable Development Goal target 3.4 [41].

This study revealed that adhering to the healthiest lifestyles was connected with a 4% to 33% decrease in the risk of cancer case fatality when compared to individuals exhibiting the lowest level of preventive indicators, as indicated by the meta-analysis [29]. However, the average follow-up duration was less than 10 years, consistent with other studies in the field [15, 42–44]. This underscores the necessity for further research with longer follow-up periods to delve into the connections between combined lifestyle factors and aspects such as quality of life, cancer recurrence, and overall survival among cancer patients.

While this study focused on primary prevention indicators, it is important to acknowledge the complementary role of cancer screening in influencing incidence rates. Screening programs, particularly for cancers such as thyroid, breast, and colorectal, can lead to earlier detection, potentially inflating incidence rates in regions with higher screening coverage. For example, the widespread use of thyroid ultrasound screening in South Korea has been associated with an increased detection of small, indolent thyroid cancers [45]. This highlights the need to interpret cancer incidence rates in the context of both prevention and screening activities. Future studies should explore the interplay between prevention efforts.

This study has several limitations that should be considered when interpreting the findings. Firstly, the data on lifestyle habits relied on self-reports, which may introduce bias and inconsistency. For example, individuals often underestimate their alcohol consumption, potentially affecting the accuracy of the analysis [46]. Additionally, selection bias, such as the"sick quitter phenomenon,"may inflate the perceived benefits of moderate alcohol consumption. Furthermore, adherence to physical activity, an important factor in cancer prevention, was not evaluated due to data limitations. Numerous studies have highlighted the role of physical activity in reducing cancer risk, and its exclusion likely underestimates the influence of lifestyle behaviors on cancer outcomes. Future research should address this gap by exploring adherence to cancer prevention guidelines, including physical activity, within the Korean population.

Secondly, while this study focused on six primary prevention indicators to assess disparities in cancer mortality among cancer patients, certain cancer-specific factors were not incorporated. For example, infection-related factors such as *Helicobacter pylori* for stomach cancer and HPV for cervical cancer were excluded from the prevention index due to data unavailability. Similarly, environmental and lifestyle factors, such as second-hand smoke exposure for lung cancer and detailed dietary patterns for colorectal and pancreatic cancers, were also omitted. These exclusions may partially explain the residual disparities in cancer incidence and the case fatality. Incorporating these confounders in future research could improve the precision and applicability of prevention strategies.

Thirdly, the study did not explicitly address the role of screening programs in influencing cancer incidence rates. Screening activities, particularly for thyroid, breast, and colorectal cancers, can lead to earlier detection and potentially higher incidence rates in regions with greater screening coverage. These effects were not accounted for in the current analysis, potentially confounding the observed associations. Future studies should incorporate screening data to provide a more comprehensive understanding of the relationship between prevention efforts, screening, and cancer incidence.

Fourthly, the intercorrelations between primary prevention indicators, such as obesity, hypertension, and diabetes, present a methodological challenge in isolating their individual effects on cancer outcomes. Although multicollinearity was assessed and controlled statistically, the complex interplay between these factors warrants further investigation in future studies.

Fifthly, the latency period between prevention efforts and cancer outcomes was not fully addressed. Cancer development often spans years or even decades, and the temporal alignment between prevention indicators and cancer incidence or mortality requires additional refinement. The current analysis may not fully capture these long-term relationships, underscoring the need for future longitudinal studies with extended follow-up periods.

Moreover, the final analysis of cancer case fatality used trajectory modeling to categorize prevention indicator scores into three groups. While this approach simplified the analysis, it may have reduced the precision of the findings. Expanding the trajectory analysis to a nationwide scale and employing more detailed classifications of prevention indicator scores could enhance the understanding of how varying levels of prevention efforts impact cancer outcomes. Additionally, the study had a limited sample size at the district level, even though it was conducted in South Korea’s most densely populated region. This limitation may have reduced the statistical power of the analysis. Future research could benefit from larger datasets, particularly on a national scale, to address this issue.

Finally, variations in regional cancer registration practices and access to healthcare services may have introduced biases in cancer incidence data. Additionally, individuals who did not undergo health examinations were excluded from the study, potentially introducing bias by limiting the findings to a healthier or more health-conscious subset of the population. These limitations highlight the need for caution when generalizing the results and underscore the importance of further studies to refine the methodology and address these shortcomings.

## Conclusions

In conclusion, promoting the adoption of comprehensive healthy lifestyles is crucial for effectively preventing cancer-related mortality. However, the percentage of individuals adhering to these healthy practices remains low in many regions. Therefore, establishing an environment that fosters behavior modifications should be prioritized in public health efforts. Further research is imperative, including the incorporation of all possible comprehensive preventive indicators while adjusting for cancer- and site-specific confounding factors. Additionally, more evidence among cancer survivors and populations in low- and middle-income regions is needed to gain a better understanding of the impact of healthy lifestyles on cancer outcomes.

## Supplementary Information

Additional file 1. Table S1. List and ICD-10 codes for disease. Table S2. List and definition of primary prevention indicators. Table S3. Number of cancer patients by prevention index level, gender, and SEER stage during 2010–2020 in Gyeonggi Province. Table S4. Distribution of cancer indicators according to the sub domains. Table S5. The results from the trajectory modeling for prevention indicatorsduring 2010–2018 in Gyeonggi Province. Table S6. The results from the trajectory modeling for cancer incidence rateduring 2010–2020 in Gyeonggi Province. Fig. S1 Graphs depicting the comprehensive scores of primary preventive indicators, levels, and cancer incidence rates during 2010–2020 in Gyeonggi Province.

## Data Availability

The data presented in this study are available from the corresponding author on reasonable request.

## Abbreviations

IARC: International Agency for Research on Cancer
ICD: International Classification of Diseases
KCHS: The Korea Community Health Survey
KDCA: Korea Disease Control and Prevention Agency
NHID: National Health Information Database
NHIS: National Health Insurance Service
