# Supplementary material for: Connection between trajectory of primary cancer monitoring indicators and mortality after cancer in South Korea
Source: BMC Med. 2025 May 30;23:320. doi: 10.1186/s12916-025-04121-y (PMC12124092; doi:10.1186/s12916-025-04121-y)

**Additional file 1**

**Additional file 1: Table S1**. List and ICD-10 codes for disease

| **Disease Type** | **ICD-10 Code** |
| --- | --- |
| **Stomach Cancer** | C16 |
| **Colorectal Cancer** | C18–C20 |
| **Liver Cancer** | C22 |
| **Pancreatic Cancer** | C25 |
| **Lung Cancer** | C33–C34 |
| **Breast Cancer** | C50 |
| **Cervical Cancer** | C53 |
| **Prostate Cancer** | C61 |
| **Thyroid Cancer** | C73 |
| **Leukemia** | C91–C95 |
| **Non-Hodgkin Lymphoma** | C82–C86, C96 |
| **Hypertension** | I10–I15 |
| **Diabetes** | E10–E14 |

**Additional file 1: Table S2**. List and definition of primary prevention indicators

| **No** | **Indicator** | **Definition** |
| --- | --- | --- |
| 1 | Obesity prevalence | The percentage of people with a Body Mass Index (BMI) of 25kg/m2 or higher among the total population. |
| 2 | Physical  activity rate | The percentage of people who regularly engage in moderate or higher intensity exercise among the total population.  The number of people who have engaged in moderate or higher intensity physical activity in the past week.  The number of people who have engaged in vigorous physical activity for at least 20 minutes per day, 3 or more days a week, or engaged in moderate intensity physical activity for at least 30 minutes per day, 5 or more days a week in the past week.  Definition of vigorous physical activity: activities such as running (jogging), hiking, cycling at a fast pace, fast swimming, soccer, basketball, jump rope, squash, singles tennis, carrying heavy objects as part of occupation or physical activity.  Definition of moderate intensity physical activity: activities such as slow swimming, doubles tennis, volleyball, badminton, table tennis, carrying light objects as part of occupation or physical activity. |
| 3 | High-risk alcohol drinking rate | The percentage of people in the total population who fall under high-risk drinking. Definition of high-risk drinking: Individuals who have consumed alcohol two or more times per week in the past year and have an average alcohol intake of at least 7 drinks for males or 5 drinks for females per occasion. |
| 4 | Hypertension diagnosis rate | The percentage of people diagnosed with hypertension by doctors relative to the population covered by that healthcare jurisdiction. |
| 5 | Diabetes diagnosis rate | The percentage of people diagnosed with diabetes by doctors relative to the population covered by that healthcare jurisdiction. |
| 6 | Average daily per capita  smoking amount | The average number of cigarette packs smoked per day by current smokers. |
| 7 | Smoking rate | The percentage of people who currently smoke among those who have smoked more than 5 packs (100 cigarettes) in their lifetime. |

**Additional file 1: Table S3.** Number of cancer patients by prevention index level (PI level), gender, and SEER stage during 2010-2020 in Gyeonggi Province

|  | **PI level = High** | | | | | | | | | |
| --- | --- | --- | --- | --- | --- | --- | --- | --- | --- | --- |
|  | **Male** (N=12,450) | | | | | | **Female** (N=15,543) | | | |
|  | SEER stage | | | | | | SEER stage | | | |
|  | Localized | Regional | | Distant | Unknown | | Localized | Regional | Distant | Unknown |
| **Thyroid cancer** | 637 | 1,285 | | 18 | 160 | | 2,096 | 2,953 | 17 | 499 |
| **Colorectal cancer** | 871 | 871 | | 303 | 127 | | 621 | 682 | 250 | 85 |
| **Leukemia** | 0 | 0 | | 285 | 15 | | 0 | 0 | 220 | 13 |
| **Non-Hodgkin lymphomas** | 128 | 66 | | 180 | 101 | | 90 | 35 | 128 | 88 |
| **Stomach cancer** | 1,770 | 569 | | 252 | 118 | | 855 | 333 | 163 | 77 |
| **Breast cancer** | 4 | 9 | | 1 | 0 | | 2,712 | 1,332 | 198 | 131 |
| **Cervical cancer** | - | - | | - | - | | 275 | 115 | 46 | 54 |
| **Prostate cancer** | 1,306 | 440 | | 133 | 436 | | - | - | - | - |
| **Pancreatic cancer** | 52 | 142 | | 214 | 56 | | 50 | 110 | 162 | 52 |
| **Lung cancer** | 504 | 533 | | 669 | 195 | | 396 | 253 | 366 | 86 |
| **Total** | 5,272 | 3,915 | | 2,055 | 1,208 | | 7,095 | 5,813 | 1,550 | 1,085 |
|  | **PI level = Medium** | | | | | | | | | |
|  | **Male** (N=40,527) | | | | | **Female** (N=49,103) | | | | |
|  | SEER stage | | | | | SEER stage | | | | |
|  | Localized | Regional | | Distant | Unknown | | Localized | Regional | Distant | Unknown |
| **Thyroid cancer** | 1,503 | 2,614 | | 46 | 271 | | 6,801 | 8,455 | 71 | 1,175 |
| **Colorectal cancer** | 3,674 | 3,319 | | 1,313 | 460 | | 2,261 | 2,364 | 949 | 350 |
| **Leukemia** | 0 | 0 | 959 | | 61 | | 0 | 0 | 727 | 43 |
| **Non-Hodgkin lymphomas** | 426 | 225 | 554 | | 198 | | 318 | 164 | 383 | 156 |
| **Stomach cancer** | 6,694 | 2,043 | 1,010 | | 479 | | 3,038 | 961 | 565 | 277 |
| **Breast cancer** | 23 | 14 | 4 | | 1 | | 7,744 | 4,486 | 581 | 370 |
| **Cervical cancer** | - | - | - | | - | | 1,150 | 528 | 166 | 150 |
| **Prostate cancer** | 3,334 | 1,169 | 534 | | 599 | | - | - | - | - |
| **Pancreatic cancer** | 161 | 539 | 817 | | 148 | | 166 | 440 | 589 | 206 |
| **Lung cancer** | 1,463 | 2,026 | 3,200 | | 646 | | 996 | 756 | 1,420 | 297 |
| **Total** | 17,278 | 11,949 | 8,437 | | 2,863 | | 22,474 | 18,154 | 5,451 | 3,024 |
|  | **PI level = Low** | | | | | | | | | |
|  | **Male** (N=84,475) | | | | | | **Female** (N=91,972) | | | |
|  | SEER stage | | | | | | SEER stage | | | |
|  | Localized | Regional | | Distant | Unknown | | Localized | Regional | Distant | Unknown |
| **Thyroid cancer** | 2,587 | 4,380 | | 83 | 476 | | 12,306 | 14,831 | 163 | 1,643 |
| **Colorectal cancer** | 7,077 | 7,429 | | 2,771 | 972 | | 4,149 | 4,982 | 1,897 | 723 |
| **Leukemia** | 0 | 0 | | 1,978 | 101 | | 0 | 0 | 1,450 | 66 |
| **Non-Hodgkin lymphomas** | 851 | 343 | | 1,151 | 392 | | 582 | 265 | 825 | 280 |
| **Stomach cancer** | 14,038 | 4,989 | | 2,450 | 1,012 | | 6,261 | 2,268 | 1,140 | 579 |
| **Breast cancer** | 47 | 41 | | 6 | 1 | | 13,258 | 7,827 | 1,120 | 686 |
| **Cervical cancer** | - | - | | - | - | | 2,488 | 1,159 | 379 | 313 |
| **Prostate cancer** | 6,257 | 2,274 | | 989 | 1,303 | | - | - | - | - |
| **Pancreatic cancer** | 384 | 1,008 | | 1,532 | 356 | | 427 | 907 | 1,219 | 399 |
| **Lung cancer** | 3,401 | 5,026 | | 7,273 | 1,497 | | 2,007 | 1,748 | 3,028 | 597 |
| **Total** | 34,642 | 25,490 | | 18,233 | 6,110 | | 41,478 | 33,987 | 11,221 | 5,286 |

The selection of primary prevention indicators for this study was drew upon the findings of our own previous research by the scoping review (Rajaguru et al. 2022), which explored population-centered indicators throughout the cancer care continuum. The process involved a comprehensive review of 61 potential indicators, which were derived from a systematic search and assessment of the literature. The reviewed indicators were categorized into five main dimensions: classification, participants, domains, subdomains, and measurement methods. This ensured a holistic approach to identifying indicators that addressed behavioral, environmental, and systemic factors relevant to cancer prevention. Through multidisciplinary consultations involving researchers, policymakers, and clinicians, the indicators were evaluated for relevance, feasibility, and potential impact on cancer care. Key considerations included their ability to represent modifiable risk factors and align with community-based cancer prevention strategies​. From this pool of 61 indicators, six were ultimately selected for their strong relevance to modifiable risk factors for cancer incidence: obesity prevalence, high-risk alcohol consumption, smoking prevalence, average daily smoking amount, hypertension prevalence, and diabetes prevalence. These indicators were chosen based on their significant contribution to cancer risk as evidenced in prior studies and their practical applicability for regional monitoring and intervention​.

**Additional file 1: Table S4.** Distribution of cancer indicators according to the sub domains

| **Classification/ subdomains** | **Primary Prevention** |
| --- | --- |
| **Smoking** | 14 |
| **Alcohol** | 14 |
| **Nutrition** | 3 |
| **Obesity** | 3 |
| **Physical activity** | 4 |
| **Research & investment** | 8 |
| **High risk for infection** | 3 |
| **High risk for chronic disease** | 1 |
| **High risk for occupation environment** | 4 |
| **Health care system** | 1 |
| **Health professionals** | 5 |
| **Vaccine/immunization** | 1 |
| **Incidence** | – |
| **Diagnosis** | – |
| **Prevalence** | – |
| **General health checkup** | – |
| **Facilities** | – |
| **Patient-centered** | – |
| **Treatment/consultation/ interruption rate** | – |
| **Treatment plan & record** | – |
| **Specific procedure and treatment** | – |
| **Health expenditure** | – |
| **Survival/mortality** | – |
| **Total** | 61 |

**Additional file 1: Table S5.** The results from the trajectory modeling for prevention indicators (PI) during 2010-2018 in Gyeonggi Province

| Maximum Likelihood Estimates(Obesity prevalence) | | | | | |
| --- | --- | --- | --- | --- | --- |
| Group | Parameter | Estimate | Standard Error | T | P-value |
| Level 1 | Intercept | 24.22 | 17176.83 | 0.001 | 0.99 |
|  | Linear | 404.03 | 49.23 | 8.20 | 0.00 |
|  | Quadratic | -0.40 | 0.016 | -25.02 | 0.00 |
|  | Cubic | 0.0001 | 0.00001 | 13.62 | 0.00 |
| Level 2 | Intercept | -2750.48 | 779.73 | -3.52 | 0.0005 |
|  | Linear | 1.37 | 0.38 | 3.55 | 0.0004 |
| Group Membership |  |  |  |  |  |
| Level 1 | (%) | 95.21 | 3.16 | 30.05 | 0.00 |
| Level 2 | (%) | 4.78 | 3.167 | 1.510 | 0.13 |

| Maximum Likelihood Estimates(high-risk drinking rate) | | | | | |
| --- | --- | --- | --- | --- | --- |
| Group | Parameter | Estimate | Standard Error | T | P-value |
| Level 3 | Intercept | 13.56 | 38285.27 | 0.000 | 0.99 |
|  | Linear | -414.61 | 26.58 | -15.59 | 0.00 |
|  | Quadratic | 0.41 | 0.043 | 9.37 | 0.00 |
|  | Cubic | -0.0001 | 0.00001 | -17.57 | 0.00 |
| Level 2 | Intercept | -665.88 | 179.50 | -3.71 | 0.0002 |
|  | Linear | 0.33 | 0.08 | 3.79 | 0.0002 |
| Level 1 | Intercept | -761.19 | 166.32 | -4.57 | 0.00 |
|  | Linear | 0.38 | 0.08 | 4.69 | 0.00 |
| Group Memberships |  |  |  |  |  |
| Level 3 | (%) | 4.87 | 3.46 | 1.41 | 0.1594 |
| Level 2 | (%) | 44.81 | 9.05 | 4.949 | 0.00 |
| Level 1 | (%) | 50.30 | 9.15 | 5.493 | 0.00 |

| Maximum Likelihood Estimates(hypertension diagnosis rate) | | | | | |
| --- | --- | --- | --- | --- | --- |
| Group | Parameter | Estimate | Standard Error | T | P-value |
| Level 3 | Intercept | 14.59 | 11578.81 | 0.001 | 0.99 |
|  | Linear | 60.47 | 30.33 | 1.99 | 0.04 |
|  | Quadratic | -0.06 | 0.01 | -4.56 | 0.00 |
|  | Cubic | 0.00001 | 0.00 | 7.30 | 0.00 |
| Level 2 | Intercept | 15.63 | 0.14 | 104.99 | 0.00 |
| Level 1 | Intercept | -276.66 | 82.58 | -3.35 | 0.0009 |
|  | Linear | 0.14 | 0.04 | 3.55 | 0.0004 |
| Group Membership |  |  |  |  |  |
| Level 3 | (%) | 7.85 | 4.20 | 1.86 | 0.06 |
| Level 2 | (%) | 39.94 | 12.37 | 3.22 | 0.001 |
| Level 1 | (%) | 52.19 | 12.47 | 4.18 | 0.00 |

| Maximum Likelihood Estimates(diabetes diagnosis rate) | | | | | |
| --- | --- | --- | --- | --- | --- |
| Group | Parameter | Estimate | Standard Error | T | P-value |
| Level 3 | Intercept | 5.22 | 0.10 | 52.08 | 0.00 |
| Level 2 | Intercept | 6.30 | 0.21 | 29.63 | 0.00 |
| Level 1 | Intercept | -263.63 | 107.52 | -2.45 | 0.01 |
|  | Linear | 0.13 | 0.05 | 2.51 | 0.01 |
| Group Membership |  |  |  |  |  |
| Level 3 | (%) | 23.77 | 6.90 | 3.44 | 0.0006 |
| Level 2 | (%) | 29.32 | 18.65 | 1.57 | 0.11 |
| Level 1 | (%) | 46.90 | 19.38 | 2.42 | 0.01 |

| Maximum Likelihood Estimates(average daily per capita smoking amount) | | | | | |
| --- | --- | --- | --- | --- | --- |
| Group | Parameter | Estimate | Standard Error | T | P-value |
| Level 2 | Intercept | 14.40 | 10593.00 | 0.001 | 0.99 |
|  | Linear | 64.14 | 13.39 | 4.78 | 0.00 |
|  | Quadratic | -0.06 | 0.00 | -27.15 | 0.00 |
|  | Cubic | 0.00002 | 0.00 | 7.21 | 0.00 |
| Level 1 | Intercept | 470.60 | 36.68 | 12.82 | 0.00 |
|  | Linear | -0.22 | 0.01 | -12.41 | 0.00 |
| Group Membership |  |  |  |  |  |
| Level 2 | (%) | 11.95 | 4.90 | 2.43 | 0.01 |
| Level 1 | (%) | 88.04 | 4.90 | 17.94 | 0.00 |

| Maximum Likelihood Estimates(smoking rate) | | | | | |
| --- | --- | --- | --- | --- | --- |
| Group | Parameter | Estimate | Standard Error | T | P-value |
| Level 2 | Intercept | 21.21 | 31468.56 | 0.001 | 0.99 |
|  | Linear | 156.06 | 46.41 | 3.36 | 0.0009 |
|  | Quadratic | -0.15 | 0.008 | -17.68 | 0.00 |
|  | Cubic | 0.00004 | 0.00 | 8.16 | 0.00 |
| Level 1 | Intercept | 1397.20 | 112.43 | 12.42 | 0.00 |
|  | Linear | -0.68 | 0.05 | -12.21 | 0.00 |
| Group Membership |  |  |  |  |  |
| Level 2 | (%) | 25.08 | 6.78 | 3.69 | 0.0003 |
| Level 1 | (%) | 74.91 | 6.78 | 11.03 | 0.00 |

**Additional file 1: Table S6.** The results from the trajectory modeling for cancer incidence rate (CI) during 2010-2020 in Gyeonggi Province

| Maximum Likelihood Estimates(cancer incidence rate) | | | | | |
| --- | --- | --- | --- | --- | --- |
| Group | Parameter | Estimate | Standard Error | T | P-value |
| Level 3 | Intercept | 182.33 | 483300.00 | 0.00 | 1.00 |
|  | Linear | 964.71 | 933.39 | 1.03 | 0.30 |
|  | Quadratic | -0.96 | 0.27 | -3.58 | 0.00 |
|  | Cubic | 0.00 | 0.00 | 2.74 | 0.01 |
| Level 2 | Intercept | -10402.32 | 1162.75 | -8.95 | 0.00 |
|  | Linear | 5.28 | 0.58 | 9.14 | 0.00 |
| Level 1 | Intercept | -6374.56 | 2682.47 | -2.38 | 0.02 |
|  | Linear | 3.32 | 1.33 | 2.49 | 0.01 |
| Group Membership |  |  |  |  |  |
| Level 3 | (%) | 35.40 | 8.11 | 4.36 | 0.00 |
| Level 2 | (%) | 55.07 | 7.29 | 7.56 | 0.00 |
| Level 1 | (%) | 9.52 | 4.36 | 2.18 | 0.03 |

**Additional file 1: Fig. S1**. Graphs depicting the comprehensive scores of primary preventive indicators, levels,
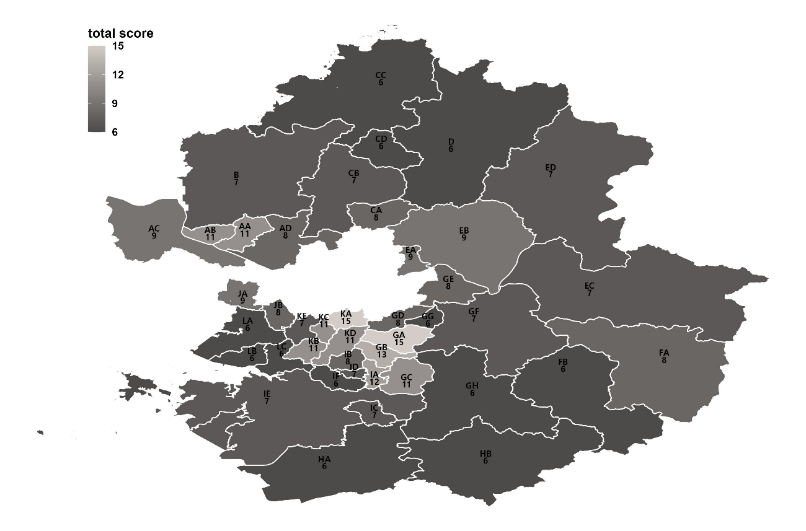
and cancer incidence rates during 2010-2020 in Gyeonggi Province


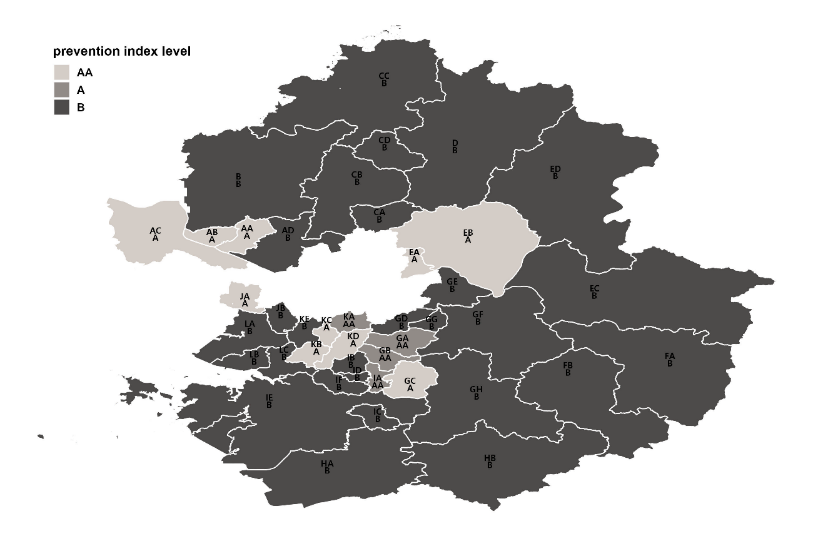


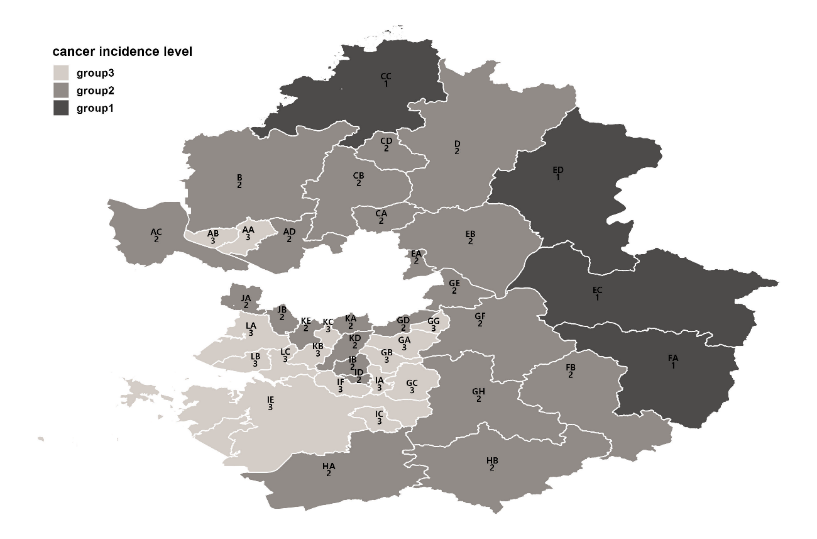

Supplement: Supplementary file 1 — Additional file 1. Table S1. List and ICD-10 codes for disease. Table S2. List and definition of primary prevention indicators. Table S3. Number of cancer patients by prevention index level, gender, and SEER stage during 2010–2020 in Gyeonggi Province. Table S4. Distribution of cancer indicators according to the sub domains. Table S5. The results from the trajectory modeling for prevention indicatorsduring 2010–2018 in Gyeonggi Province. Table S6. The results from the trajectory modeling for cancer incidence rateduring 2010–2020 in Gyeonggi Province. Fig. S1 Graphs depicting the comprehensive scores of primary preventive indicators, levels, and cancer incidence rates during 2010–2020 in Gyeonggi Province. [file 12916_2025_4121_MOESM1_ESM.docx]
